# Supplementary material for: Predictors of maternal HIV acquisition during pregnancy and lactation in sub-Saharan Africa: A systematic review and narrative synthesis
Source: PLoS One. 2024 Dec 3;19(12):e0314747. doi: 10.1371/journal.pone.0314747 (PMC11614209; doi:10.1371/journal.pone.0314747)
Supplement: S1 Table — (DOCX) [file pone.0314747.s001.docx]

Table S1. Database search strategy

| **PubMed** | |
| --- | --- |
| Number | Query |
| 1 | Pregnancy[mesh:noexp] OR pregnancy[tw] OR pregnant[tw] OR antenatal[tw] OR ante natal[tw] OR prenatal care[mesh] OR prenatal[tw] OR perinatal care[mesh] OR perinatal[tw] OR antepartum[tw] OR ante partum[tw] OR postnatal care[mesh] OR postnatal[tw] OR post natal[tw] OR postpartum period[mesh] OR postpartum[tw] OR post partum[tw] OR puerperium[tw] OR puerperal[tw] OR lactation[tw] OR lactating[tw] OR lactate[tw] OR breastfeed[tw] OR breastfeeding[tw] OR breast feeding[tw] OR gravidity[mesh] |
| 2 | Africa South of the Sahara[mesh] OR sub-Saharan Africa[tw] OR Subsaharan Africa[tw] OR sub Saharan Africa[tw] OR sub-Sahara[tw] OR Central Africa[tw] OR Southern Africa[tw] OR East Africa[tw] OR Eastern Africa[tw] OR West Africa[tw] OR Western Africa[tw] OR Angola[tw] OR Benin[tw] OR Botswana[tw] OR Burkina Faso[tw] OR Burundi[tw] OR Cameroon[tw] OR Cape Verde[tw] OR Central African Republic[tw] OR Chad[tw] OR Comoros[tw] OR Congo[tw] OR Côte d'Ivoire[tw] OR Ivory Coast[tw] OR Djibouti[tw] OR DRC[tw] OR Eritrea[tw] OR Ethiopia[tw] OR Gabon[tw] OR Gambia[tw] OR Ghana[tw] OR Guinea[tw] OR Kenya[tw] OR Lesotho[tw] OR Liberia[tw] OR Madagascar[tw] OR Malawi[tw] OR Mali[tw] OR Mauritania[tw] OR Mauritius[tw] OR Mayotte[tw] OR Mozambique[tw] OR Mocambique[tw] OR Namibia[tw] OR Niger[tw] OR Nigeria[tw] OR Réunion[tw] OR Rwanda[tw] OR Sahel[tw] OR Sao Tome and Principe[tw] OR Senegal[tw] OR Seychelles[tw] OR Sierra Leone[tw] OR Somalia[tw] OR South Africa[tw] OR RSA[tw] OR Sudan[tw] OR Swaziland[tw] OR Tanzania[tw] OR Togo[tw] OR Uganda[tw] OR Zambia[tw] OR Zimbabwe[tw] |
| 3 | HIV[mesh] OR HIV[tw] OR Human Immunodeficiency Virus[tw] OR Human Immunodeficiency Viruses[tw] OR HIV/AIDS[tw] OR HIV Infections[mesh:noexp] OR HIV infection[tw] OR HIV infections[tw] OR HIV Seropositivity[tw] OR HIV Seropositivities[tw] OR HIV seroconversion[tw] |
| 4 | Protective Factors[mesh] OR protective factor[tw] OR protective factors[tw] OR incidence[tw] OR incident[tw] OR acquisition[tw] OR acquiring[tw] OR acquired[tw] OR acquire[tw] OR acute[tw] OR risk [tw] OR risk factor[tw] OR risk factors[tw] |
| 5 | ("1980/01/01"[Date - Publication] : "2024/03/31"[Date - Publication]) |
| 6 | 1 AND 2 AND 3 AND 4 AND 5 |
| **Embase** | |
| Number | Query |
| 1 | (‘pregnancy’/de OR ‘prenatal care’/de OR ‘perinatal care’/de OR ‘postnatal care’/de OR ‘puerperium’/de OR ‘lactation’/de (pregnancy OR pregnant OR prenatal OR antenatal OR ‘ante natal’ OR ‘ante-natal’ OR perinatal OR antepartum OR ‘ante partum’ OR ‘ante-partum’ OR postnatal OR ‘post natal’ OR ‘post-natal’ OR puerperium OR puerperal OR postpartum OR ‘post partum’ OR ‘post-partum’ OR lactation OR lactating OR lactate OR breastfeed OR breastfeeding OR ‘breast feeding’ OR ‘breast-feeding’ OR gravidity):ab,ti) |
| 2 | (‘Africa south of the Sahara’/exp OR (‘sub-Saharan Africa’ OR ‘Subsaharan Africa’ OR ‘sub Saharan Africa’ OR ‘sub-Sahara’ OR ‘Central Africa’ OR ‘Southern Africa’ OR ‘East Africa’ OR ‘Eastern Africa’ OR ‘West Africa’ OR ‘Western Africa’ OR Angola OR Benin OR Botswana OR ‘Burkina Faso’ OR Burundi OR Cameroon OR ‘Cape Verde’ OR ‘Central African Republic’ OR Chad OR Comoros OR Congo OR ‘Cote d Ivoire’ OR ‘Ivory Coast’ OR Djibouti OR DRC OR Eritrea OR Ethiopia OR Gabon OR Gambia OR Ghana OR Guinea OR Kenya OR Lesotho OR Liberia OR Madagascar OR Malawi OR Mali OR Mauritania OR Mauritius OR Mayotte OR Mozambique OR Mocambique OR Namibia OR Niger OR Nigeria OR Réunion OR Rwanda OR Sahel OR ‘Sao Tome and Principe’ OR Senegal OR Seychelles OR ‘Sierra Leone’ OR Somalia OR ‘South Africa’ OR RSA OR Sudan OR Swaziland OR Tanzania OR Togo OR Uganda OR Zambia OR Zimbabwe):ab,ti) |
| 3 | (‘human immunodeficiency virus infection’/de OR ‘acute HIV infection’/de OR ‘Human immunodeficiency virus 1 infection’/de OR ‘Human immunodeficiency virus 2 infection’/de OR (HIV OR ‘Human Immunodeficiency Virus’ OR ‘Human Immunodeficiency Viruses’ OR ‘HIV infection’ OR ‘HIV infections’ OR ‘hiv seropositivity’ OR ‘HIV seropositivities’ OR ‘hiv seroconversion’ OR ‘HIV/AIDS’):ab,ti) |
| 4 | (‘incidence’/de OR ‘risk factor’/de OR (incidence OR incident OR acquisition OR acquiring OR acquired OR acquire OR acute OR ‘risk factor’ OR ‘risk factors’ OR ‘protective factor’ OR ‘protective factors’ OR ‘risk’):ab,ti) |
| 5 | 01/01/1980 to present (31/03/2024) |
| 6 | #1 AND #2 AND #3 AND #4 AND #5 |
| 7 | #1 AND #2 AND #3 AND #4 AND #5 AND [embase]/lim |
| **PsycInfo** | |
| Number | Query |
| 1 | DE (“pregnancy” OR “prenatal care” OR DE “perinatal period” OR DE “antepartum period” OR DE “postnatal period” OR DE “lactation”) OR TI (“pregnancy” OR “prenatal” OR “pre natal” OR “pre-natal” OR “antenatal” OR “ante natal” OR “perinatal” OR “antepartum” OR “ante partum” OR “ante-partum” OR “postnatal” OR “post natal” OR “post-natal” OR “postpartum” OR “post partum” OR “post-partum” OR “puerperium” OR “puerperal” OR “lactation” OR “lactating” OR “lactate” OR “breastfeed” OR “breastfeeding” OR “breast feeding” OR “breast-feeding” OR “gravidity”) OR AB (“pregnancy” OR “prenatal” OR “pre natal” OR “pre-natal” OR “antenatal” OR “ante natal” “perinatal” OR “antepartum” OR “ante partum” OR “ante-partum” OR “postnatal” OR “post natal” OR “post-natal” OR “postpartum” OR “post partum” OR “post-partum” OR “puerperium” OR “puerperal” OR “lactation” OR “lactating” OR “lactate” OR “breastfeed” OR “breastfeeding” OR “breast feeding” OR “breast-feeding” OR “gravidity”) |
| 2 | TI (“sub-Saharan Africa” OR “Subsaharan Africa” OR “sub Saharan Africa” OR “sub-Sahara” OR “Central Africa” OR “Southern Africa” OR “East Africa” OR “Eastern Africa” OR “West Africa” OR “Western Africa” OR “Angola” OR “Benin” OR “Botswana” OR “Burkina Faso” OR “Burundi” OR “Cameroon” OR “Cape Verde” OR “Central African Republic” OR “Chad” OR “Comoros” OR “Congo” OR “Cote d Ivoire” OR “Ivory Coast” OR “Djibouti” OR “DRC” OR “Eritrea” OR “Ethiopia” OR “Gabon” OR “Gambia” OR “Ghana” OR “Guinea” OR “Kenya” OR “Lesotho” OR “Liberia” OR “Madagascar” OR “Malawi” OR “Mali” OR “Mauritania” OR “Mauritius” OR “Mayotte” OR “Mozambique” OR “Mocambique” OR “Namibia” OR “Niger” OR “Nigeria” OR “Réunion” OR “Rwanda” OR “Sahel” OR “Sao Tome and Principe” OR “Senegal” OR “Seychelles” OR “Sierra Leone” OR “Somalia” OR “South Africa” OR “RSA” OR “Sudan” OR “Swaziland” OR “Tanzania” OR “Togo” OR “Uganda” OR “Zambia” OR “Zimbabwe”) OR AB (“sub-Saharan Africa” OR “Subsaharan Africa” OR “sub Saharan Africa” OR “sub-Sahara” OR “Central Africa” OR “Southern Africa” OR “East Africa” OR “Eastern Africa” OR “West Africa” OR “Western Africa” OR “Angola” OR “Benin” OR “Botswana” OR “Burkina Faso” OR “Burundi” OR “Cameroon” OR “Cape Verde” OR “Central African Republic” OR “Chad” OR “Comoros” OR “Congo” OR “Cote d Ivoire” OR “Ivory Coast” OR “Djibouti” OR “DRC” OR “Eritrea” OR “Ethiopia” OR “Gabon” OR “Gambia” OR “Ghana” OR “Guinea” OR “Kenya” OR “Lesotho” OR “Liberia” OR “Madagascar” OR “Malawi” OR “Mali” OR “Mauritania” OR “Mauritius” OR “Mayotte” OR “Mozambique” OR “Mocambique” OR “Namibia” OR “Niger” OR “Nigeria” OR “Réunion” OR “Rwanda” OR “Sahel” OR “Sao Tome and Principe” OR “Senegal” OR “Seychelles” OR “Sierra Leone” OR “Somalia” OR “South Africa” OR “RSA” OR “Sudan” OR “Swaziland” OR “Tanzania” OR “Togo” OR “Uganda” OR “Zambia” OR “Zimbabwe”) |
| 3 | DE (“HIV”) OR TI (“HIV” OR “human immunodeficiency virus” OR “human immunodeficiency viruses” OR “HIV infection” OR “HIV infections” OR “HIV seropositivity” OR “HIV Seropositivities” OR “HIV/AIDS”) OR AB (“HIV” OR “human immunodeficiency virus” OR “human immunodeficiency viruses” OR “HIV infection” OR “HIV infections” OR “HIV seropositivity” OR “HIV Seropositivities” OR “HIV seroconversion” OR “HIV/AIDS”) |
| 4 | DE(“risk factors” OR “protective factors”) OR TI(“incidence” OR “incident” OR “acquisition” OR “acquiring” OR “acquired” OR “acute” OR “risk factor” OR “risk factors” OR “protective factor” OR “protective factors” OR “risk”) OR AB(“incidence” OR “incident” OR “acquisition” OR “acquiring” OR “acquired” OR “acute” OR “risk factor” OR “risk factors” OR “protective factor” OR “protective factors” OR “risk”) |
| 5 | (DT 19800101-20240331) |
| 6 | S1 AND S2 AND S3 AND S4 AND S5 |
| **Cochrane Library** | |
| Number | Query |
| 1 | MeSH descriptor: [Pregnancy] this term only |
| 2 | MeSH descriptor: [Prenatal Care] this term only |
| 3 | MeSH descriptor: [Perinatal Care] 1 tree(s) exploded |
| 4 | MeSH descriptor: [Postnatal Care] 2 tree(s) exploded |
| 5 | MeSH descriptor: [Postpartum Period] explode all trees |
| 6 | MeSH descriptor: [Lactation] this term only |
| 7 | MeSH descriptor: [Gravidity] explode all trees |
| 8 | “pregnancy” OR “prenatal” OR “pre natal” OR “pre-natal” OR “antenatal” OR “ante natal” OR “perinatal” OR “antepartum” OR “ante partum” OR “ante-partum” OR “postnatal” OR “post natal” OR “post-natal” OR “postpartum” OR “post partum” OR “post-partum” OR “puerperium” OR “puerperal” OR “lactation” OR “lactating” OR “lactate” OR “breastfeed” OR “breastfeeding” OR “breast feeding” OR “breast-feeding” OR “gravidity”:ti,ab,kw (Word variations have been searched) |
| 9 | #1 OR #2 OR #3 OR #4 OR #5 OR #6 OR #7 OR #8 |
| 10 | MeSH descriptor: [Africa South of the Sahara] explode all trees |
| 11 | “sub-Saharan Africa” OR “Subsaharan Africa” OR “sub Saharan Africa” OR “sub-Sahara” OR “Central Africa” OR “Southern Africa” OR “East Africa” OR “Eastern Africa” OR “West Africa” OR “Western Africa” OR “Angola” OR “Benin” OR “Botswana” OR “Burkina Faso” OR “Burundi” OR “Cameroon” OR “Cape Verde” OR “Central African Republic” OR “Chad” OR “Comoros” OR “Congo” OR “Cote d Ivoire” OR “Ivory Coast” OR “Djibouti” OR “DRC” OR “Eritrea” OR “Ethiopia” OR “Gabon” OR “Gambia” OR “Ghana” OR “Guinea” OR “Kenya” OR “Lesotho” OR “Liberia” OR “Madagascar” OR “Malawi” OR “Mali” OR “Mauritania” OR “Mauritius” OR “Mayotte” OR “Mozambique” OR “Mocambique” OR “Namibia” OR “Niger” OR “Nigeria” OR “Réunion” OR “Rwanda” OR “Sahel” OR “Sao Tome and Principe” OR “Senegal” OR “Seychelles” OR “Sierra Leone” OR “Somalia” OR “South Africa” OR “RSA” OR “Sudan” OR “Swaziland” OR “Tanzania” OR “Togo” OR “Uganda” OR “Zambia” OR “Zimbabwe”:ti,ab,kw (Word variations have been searched) |
| 12 | #10 OR #11 |
| 13 | MeSH descriptor: [HIV] this term only |
| 14 | MeSH descriptor: [HIV Infections] this term only |
| 15 | “HIV” OR “human immunodeficiency virus” OR “human immunodeficiency viruses” OR “HIV infection” OR “HIV infections” OR “HIV seropositivity” OR “HIV Seropositivities” OR “HIV seroconversion” OR “HIV/AIDS”:ti,ab,kw (Word variations have been searched) |
| 16 | #13 OR #14 OR #15 |
| 17 | MeSH descriptor: [Risk Factors] explode all trees |
| 18 | MeSH descriptor: [incidence] this term only |
| 19 | “incidence” OR “incident” OR “acquisition” OR “acquiring” OR “acquired” OR “acute” OR “risk factor” OR “risk factors” OR “protective factor” OR “protective factors” OR “risk”:ti,ab,kw (Word variations have been searched) |
| 20 | #17 OR #18 OR #19 |
| 21 | #9 AND #12 AND #16 AND #20 |
| 22 | #9 AND #12 AND #16 AND #20 not in Cochrane Groups |
