# Supplementary material for: Predictors of maternal HIV acquisition during pregnancy and lactation in sub-Saharan Africa: A systematic review and narrative synthesis
Source: PLoS One. 2024 Dec 3;19(12):e0314747. doi: 10.1371/journal.pone.0314747 (PMC11614209; doi:10.1371/journal.pone.0314747)
Supplement: S2 Table — (DOCX) [file pone.0314747.s002.docx]

Table S2. Summary of predictor operationalization in each publication

| **Sociodemographic characteristics** | |
| --- | --- |
| **Age** | |
| < 21 years of age | Miotti, 1994 |
| vs. ≥ 21 years of age |  |
| 14 to 24 years of age | Machekano, 2018  Mayaphi, 2018 |
| vs. 25 to 49 years of age |  |
| 14 to 24 years of age | Fatti, 2017 |
| vs. ≥ 25 years of age |  |
| 15 to 24 years of age | Chetty, 2017  Woldesenbet, 2021 |
| vs. 25 to 49 years of age |  |
| < 25 years of age | Ortblad, 2022 |
| vs. ≥ 25 years of age |  |
| 13 to 19 years of age | Rice, 2020 |
| 20 to 24 years of age |  |
| vs. ≥ 25 years of age |  |
| 15 to 19 years of age | Gray, 2005 |
| 20 to 29 years of age |  |
| vs. ≥ 30 years of age |  |
| 15 to 24 years of age | Mbena, 2014 |
| 25 to 29 years of age |  |
| vs. ≥ 30 years of age |  |
| < 26 years of age | Egbe, 2016 |
| 26 to 35 years of age |  |
| > 35 years of age |  |
| < 20 years of age | Bulterys, 1994 |
| 20 to 22 years of age |  |
| 23 to 25 years of age |  |
| vs. 26 to 30 years of age |  |
| < 15 years of age | Mussa, 2023 |
| 15 to 24 years of age |  |
| 25 to 35 years of age |  |
| vs. ≥ 35 years of age |  |
| 15 to 19 years of age | Leroy, 1994 |
| 20 to 24 years of age |  |
| 25 to 29 years of age |  |
| vs. ≥ 30 years of age |  |
| 18 to 19 years of age | De Schacht, 2014b Humphrey, 2006 |
| 20 to 24 years of age |  |
| 25 to 29 years of age |  |
| 30 to 34 years of age |  |
| vs. ≥ 35 years of age |  |
| Continuous variable corresponding to age (years) | De Schacht, 2014a  De Schacht, 2014b  Humphrey, 2006  Kinuthia, 2015  Schumann, 2020  Taha, 1998 |
| **Marital status** | |
| Not married | Egbe, 2016  Kinuthia, 2010  Kinuthia, 2015  Mayaphi, 2018  Mbena, 2014 |
| vs. Married |  |
| Not married or cohabitating | Humphrey, 2006  le Roux, 2019  Van de Perre, 1991 |
| vs. Married or cohabitating |  |
| Single | Woldesenbet, 2021 |
| Divorced or widowed |  |
| Cohabitating |  |
| vs. Married |  |
| Single | Rice, 2020 |
| Divorced, separated, or widowed |  |
| vs. Married |  |
| Single | Mussa, 2023 |
| Divorced or widowed |  |
| vs. Married |  |
| Never married | Gray, 2005 |
| Previously married |  |
| vs. Currently married |  |
| Single | Bulterys, 1994 |
| Divorced or widowed |  |
| Common law union |  |
| vs. Married |  |
| Divorced, separated, widowed, or single | De Schacht, 2014a  De Schacht, 2014b |
| In a relationship but not cohabiting |  |
| vs. Married or cohabiting |  |
| Single | Dinh, 2015 |
| Divorced or widowed |  |
| vs. Married or cohabiting |  |
| Never married | Machekano, 2018 |
| Divorced or widowed |  |
| vs. Married or cohabiting |  |
| Divorced, widowed, or single | Schumann, 2020 |
| In a relationship but not cohabiting |  |
| vs. Married or cohabiting |  |
| **Educational attainment** | |
| Less than primary education | De Schacht, 2014a  De Schacht, 2014b |
| vs. Primary education or higher |  |
| Primary and below | Egbe, 2016  Schumann, 2020 |
| vs. Secondary and above |  |
| Less than secondary education | Kinuthia, 2010 |
| vs. Secondary education or higher |  |
| No education | Gray, 2005 |
| Primary education |  |
| vs. Secondary education or higher |  |
| Primary education/no education | Machekano, 2018  Woldesenbet, 2021 |
| Secondary education |  |
| vs. Tertiary education |  |
| Primary education or below | Mussa, 2023 |
| Secondary education or equivalent |  |
| vs. Tertiary education or equivalent |  |
| No education | Dinh, 2015 |
| Grade 1-7 |  |
| Grade 8-12 |  |
| vs. > Grade 12 |  |
| Continuous linear variable corresponding to years of schooling complete | Kinuthia, 2015 |
| **Socioeconomic status** | |
| Lowest socioeconomic status | Dinh, 2015 |
| Low socioeconomic status |  |
| vs. Average socioeconomic status |  |
| Low socioeconomic status | Schumann, 2020 |
| vs. High socioeconomic status |  |
| Does not own a TV | Kinuthia, 2010 |
| vs. Owns a TV |  |
| Does not own a gas cooker | Kinuthia, 2010 |
| vs. Owns a gas cooker |  |
| House has electricity | Taha, 1998 |
| vs. House does not have electricity |  |
| Household income (first quartile) | Humphrey, 2006 |
| Household income (second quartile) |  |
| Household income (third quartile) |  |
| Household income (missing) |  |
| vs. Household income (fourth quartile) |  |
| Employed | Egbe, 2016  De Schacht, 2014a  Kinuthia, 2010 |
| vs. Not employed |  |
| Ever had a job | De Schacht, 2014b |
| vs. Never had a job |  |
| **Polygyny (among married participants)** | |
| Polygynous marriage | De Schacht, 2014a  De Schacht, 2014b  Kinuthia, 2010  Kinuthia, 2015  Mbena, 2014 |
| vs. Monogamous marriage |  |
| **Urbanicity** | |
| Urban community/area | Mussa, 2023 |
| vs. Rural community/area |  |
| Urban residence | Mbena, 2014 |
| vs. Rural residence |  |
| Urban residence | Bulterys, 1994 |
| Peri-urban residence |  |
| Semi-rural residence |  |
| vs. Rural residence |  |
| Urban testing district | Ortblad, 2022 |
| vs. Rural testing district |  |
| Urban testing facility (public) | Schumann, 2020 |
| Urban testing facility (private) |  |
| vs. Rural testing facility (public) |  |
| **Sexual and reproductive health** | |
| **Sexually transmitted infections (STIs)** | |
| History of STIs before risk period | Kinuthia, 2015 |
| vs. No history of STIs before risk period |  |
| STIs during risk period | Bulterys, 1994 |
| vs. No STIs during risk period |  |
| STIs during index pregnancy | Schumann, 2020 |
| vs. No STIs during index pregnancy |  |
| STIs in last three months | Mayaphi, 2018 |
| vs. No STIs in last three months |  |
| Urethral discharge during risk period | Machekano, 2018 |
| vs. No history of urethral discharge during risk period |  |
| Leukorrhea in the three months before the risk period | Van de Perre, 1991 |
| vs. No leukorrhea in the three months before the risk period |  |
| Leukorrhea during risk period | Van de Perre, 1991 |
| vs. No leukorrhea during risk period |  |
| Genital ulcers during the risk period | Bulterys, 1994  Gray, 2005  Hira, 1991  Miotti, 1994  Van de Perre, 1991 |
| vs. No genital ulcers during the risk period |  |
| Genital ulcers in the three months before the risk period | Van de Perre, 1991 |
| vs. No genital ulcers in the three months before the risk period |  |
| Tested positive for chlamydia on Aptima Combo 2 assay (chlamydia/gonorrhea) | Kinuthia, 2015 |
| vs. Tested negative for chlamydia on Aptima Combo 2 assay (chlamydia/gonorrhea) |  |
| Tested positive for gonorrhea (Aptima Combo 2 assay - chlamydia/gonorrhea) | Kinuthia, 2015 |
| vs. Tested negative for gonorrhea (Aptima Combo 2 assay - chlamydia/gonorrhea) |  |
| Tested positive for gonorrhea (cervical swabs were cultured to isolate *N. gonorrhoeae*) | Taha, 1998 |
| vs. Tested negative for gonorrhea (cervical swabs were cultured to isolate *N. gonorrhoeae*) |  |
| Diagnosed with trichomoniasis using wet microscopy | Kinuthia, 2015  Taha, 1998 |
| vs. Not diagnosed with trichomoniasis using wet microscopy |  |
| Diagnosed with trichomoniasis (diagnostic approach not reported) | Miotti, 1994 |
| vs. Not diagnosed with trichomoniasis (diagnostic approach not reported) |  |
| Tested positive for syphilis on an RPR test | Kinuthia, 2015 |
| vs. Did not test positive for syphilis on RPR test |  |
| Tested positive on RPR test with confirmation via fluorescent treponemal antibody or treponema pallidum haemagglutination assay tests | Mbena, 2014  Taha, 1998 |
| vs. Did not test positive on RPR test |  |
| **Reproductive history** | |
| Primigravida | Mussa, 2023  Woldesenbet, 2021 |
| vs. Multigravida |  |
| First pregnancy | De Schacht, 2014a |
| vs. At least one prior pregnancy |  |
| < 2 prior pregnancies | Bulterys, 1994 |
| vs. ≥ 2 prior pregnancies |  |
| Primigravida | Schumann, 2020 |
| Multigravida (1) |  |
| Multigravida (2) |  |
| vs. Multigravida (3+) |  |
| 1 pregnancy | Rice, 2020 |
| 2 pregnancies |  |
| vs. 3 pregnancies |  |
| Primiparous | Humphrey, 2006  Miotti, 1994 |
| vs. Multiparous |  |
| Nulliparous | Schumann, 2020 |
| Multiparous (1) |  |
| Multiparous (2) |  |
| vs. Multiparous (3+) |  |
| Nulliparous | Mussa, 2023 |
| Multiparous |  |
| Grand-multiparous (>5) |  |
| < 2 children | Kinuthia, 2010 |
| vs. ≥ 2 children |  |
| < 3 children | De Schacht, 2014b |
| vs. ≥ 3 children |  |
| **Age at coital debut** | |
| < 17 years of age | De Schacht, 2014a  De Schacht, 2014b |
| vs. ≥ 17 years of age |  |
| < 18 years of age | Bulterys, 1994 |
| 18 to 20 years of age |  |
| vs. ≥ 21 years of age |  |
| Continuous linear variable corresponding to age at coital debut (years) | De Schacht, 2014b Kinuthia, 2015 |
| **Behaviors and knowledge** | |
| **Multiple partners** | |
| Ever had extramarital sex before risk period | De Schacht, 2014a |
| vs. Never had extramarital sex before risk period |  |
| Ever had sex with a person other than current partner before the risk period | De Schacht, 2014b |
| vs. Never had sex with a person other than current partner before the risk period |  |
| > 1 sex partners during the risk period | Gray, 2005  Machekano, 2018 |
| vs. ≤ 1 sex partners during the risk period |  |
| ≥ 3 sex partners in the past year | Schumann, 2020 |
| 2 sex partners in the past year |  |
| vs. 1 sex partner in the past year |  |
| > Had sex with someone other than baby’s father during the risk period | Bulterys, 1994 |
| vs. Did not have sex with someone other than baby’s father during the risk period |  |
| ≥ 2 sex partners during the index pregnancy | Schumann, 2020 |
| 1 sex partner during the index pregnancy |  |
| vs. 0 sex partners during the index pregnancy |  |
| > 1 current sex partner at the beginning of the risk period | Taha, 1998 |
| vs. ≤ 1 current sex partner at the beginning of the risk period |  |
| ≥ 1 new sex partners during the risk period | Humphrey, 2006 |
| Vs. No new sex partners during the risk period |  |
| Continuous variable corresponding to lifetime number of sex partners before the risk period | Kinuthia, 2015 |
| **Condom use** | |
| Consistent condom use during the risk period | Machekano, 2018 |
| vs. Inconsistent condom use during the risk period |  |
| Any condom use during the risk period | Miotti, 1994 |
| vs. No condom use during the risk period |  |
| Consistent condom use in the last year (during the risk period) | Gray, 2005 |
| Inconsistent condom use in the last year (during the risk period) |  |
| vs. No condoms use in the last year (during the risk period) |  |
| Consistent condom use (time period undefined) | Mayaphi, 2018 |
| Inconsistent condom use (time period undefined) |  |
| vs. No condom use (time period undefined) |  |
| Always uses condoms with current partner | De Schacht, 2014a  De Schacht, 2014b |
| Sometimes uses condoms with current partner |  |
| vs. Never uses condoms with current partner |  |
| Always used condoms during the index pregnancy | Schumann, 2020 |
| Sometimes used condoms during the index pregnancy |  |
| Rarely used condoms during the index pregnancy |  |
| vs. Never used condoms during the index pregnancy |  |
| **Abstinence** | |
| Sexually active in the 30 days prior to the risk period | Kinuthia, 2015 |
| vs. Not sexually active in the 30 days prior to the risk period |  |
| Reported abstaining from sex during the risk period | Machekano, 2018 |
| vs. Did not report abstaining from sex during the risk period |  |
| Reported not being sexually active during the index pregnancy | Schumann, 2020 |
| vs. Reported being sexually active during the index pregnancy |  |
| Reported not being sexually active during the risk period | Miotti, 1994 |
| vs. Reported being sexually active during the risk period |  |
| Continuous (months of abstinence during the risk period) | Humphrey, 2006 |
| **HIV risk perception** | |
| Reported concern about becoming infected with HIV | Tavengwa, 2007 |
| vs. Did not report concern about becoming infected with HIV |  |
| Felt at risk of HIV infection in the past year | Kinuthia, 2010 |
| vs. Did not feel at risk of HIV infection in the past year |  |
| High risk | De Schacht, 2014b |
| Medium risk |  |
| vs. No risk |  |
| High risk | Schumann, 2020 |
| Some risk |  |
| Very low risk |  |
| vs. No risk |  |
| **Intravaginal Drying** | |
| Ever used something to dry vagina before the risk period | De Schacht, 2014b |
| vs. Never used something to dry vagina before the risk period |  |
| Ever engaged in dry sex (i.e. sex after using a cloth to remove vaginal secretions) during the risk period | Hira, 1991 |
| vs. Never engaged in dry sex during the risk period |  |
| Any vaginal drying in the week before the risk period | Kinuthia, 2015 |
| vs. No vaginal drying in the week before the risk period |  |
| Ever used vaginal irritants for self-treatment of vaginal discharge | Miotti, 1994 |
| vs. Never used vaginal irritants for self-treatment of vaginal discharge |  |
| **Partner characteristics** | |
| **HIV status** | |
| Aware of partner’s HIV status at the start of the risk period | De Schacht, 2014a |
| vs. Not aware of partner’s HIV status at the start of the risk period |  |
| Aware of partner’s HIV status at the end of the risk period | Dinh, 2014  Egbe, 2016 |
| vs. Not aware of partner’s HIV status at the end of the risk period |  |
| Known HIV-negative at the start of the risk period | Fatti, 2017 |
| Known HIV-positive at the start of the risk period |  |
| Newly diagnosed HIV-positive at the start of the risk period |  |
| vs. Not tested or tested but unknown HIV status at the start of the risk period |  |
| Known HIV-negative at the start of the risk period | Kinuthia, 2015  Machekano, 2018 |
| Known HIV-positive at the start of the risk period |  |
| vs. Status not known at the start of the risk period |  |
| Known HIV-negative at the end of the risk period | Mayaphi, 2018 |
| Known HIV-positive at the end of the risk period |  |
| vs. Status not known at the end of the risk period |  |
| Known HIV-positive at the start of the risk period | Hira, 1991 |
| Vs. Known HIV-negative at the start of the risk period |  |
| Known HIV-positive, known and not specified, or unknown status at the end of the risk period | Schumann, 2020 |
| vs. Known HIV-negative at the end of the risk period |  |
| **Partner Travel** | |
| Partner absent for at least one continuous month | De Schacht, 2014a  De Schacht, 2014b |
| vs. Partner not absent for at least one continuous month |  |
| Partner visits town daily | Bulterys, 1994 |
| vs. Partner does not visit town daily |  |
| Partner often abroad or away from home | Schumann, 2020 |
| vs. Partner rarely or never abroad or away from home |  |
| Mobile partner | Mbena, 2014 |
| vs. Not mobile partner |  |
